# Opportune moments for task interruptions: examining the cognitive mechanisms underlying interruption-timing effects

**DOI:** 10.3389/fpsyg.2024.1465323

**Published:** 2025-01-15

**Authors:** Patricia Hirsch, Luca Moretti, Benedikt Leichtmann, Iring Koch, Verena Nitsch

**Affiliations:** ^1^Institute of Psychology, RWTH Aachen University, Aachen, Germany; ^2^Institute of Industrial Engineering and Ergonomics, RWTH Aachen University, Aachen, Germany; ^3^Department of Psychology, Ludwig-Maximilians-Universität München, Munich, Germany

**Keywords:** task interruption, interruption timing, interruption duration, chunking, resumption cost

## Abstract

**Introduction:**

Several studies showed that task interruptions at high mental workload moments are more harmful than task interruptions at low mental workload moments. In the present study, we used a theory-driven approach to define the mental workload during primary-task execution and to examine the effects of the interruption timing on primary-task performance.

**Methods:**

Participants performed a primary task comprising a pre-defined sequence of six subtasks, with task interruptions occasionally occurring before the second, third, or fourth subtasks. Critically, the subtasks were organized either in two lag-2 repetition triplets or in two lag-2 switch triplets (e.g., *A*B*A*-*C*B*C* vs. *C*B*A*-*C*A*B*). This set-up allowed us to test two predictions about the effects of interruption timing on the resumption costs (i.e., the performance in subtasks following an interruption compared to the performance in the same subtask in non-interrupted primary tasks). First, we expected task interruptions before the fourth subtask being the less detrimental due to the presumed chunking of the six subtasks into two triplets. Second, in lag-2 switch triplets, task interruptions before the second and third subtasks were predicted to result in comparable resumption costs. In contrast, in lag-2 repetition triplets, task interruptions before the third subtask were hypothesized to be more disruptive than those before the second subtask. This is because the mental workload should be higher due to the need to overcome subtask inhibition.

**Results:**

We found an interruption-timing effect with higher resumption costs for task interruptions occurring before the third subtask compared to interruptions before the second and the fourth subtasks. However, this effect did not differ across lag-2 repetition sequences and lag-2 switch sequences.

**Discussion:**

These findings are discussed from a memory perspective and a context reconstruction perspective.

## 1 Introduction

People experience task interruptions daily in numerous work environments (e.g., Baethge et al., 2015; Bellandi et al., 2018; Czerwinski et al., 2004). Commonly, we encounter task interruptions in form of e-mails, instant messages, or phone calls (Rick et al., 2024). More unusually, yet increasingly more common, workers may even be interrupted by collaborative robots. In such collaborative workplaces, the robots can increase emotional arousal and may be perceived as obstacle or hazard (e.g., Leichtmann et al., 2022, 2023), thus possibly causing task interruptions (e.g., Leichtmann et al., 2018). Although task interruptions have occasionally been shown to bring some benefits (Feldman and Greenway, 2021), they typically result in a performance decline in the interrupted task (e.g., Piątkowski et al., 2024; see Couffe and Michael, 2017; Hirsch et al., 2022; Trafton and Monk, 2007, for reviews). Thus, task interruption might negatively impact work efficiency or even lead to drastic consequences in safety-critical domains. To develop evidence-based recommendations for task-interruption management that reduce the negative effects of task interruptions, it is important to understand the cognitive mechanisms underlying their detrimental effects. To gain a deeper understanding of these mechanisms, the present study examined the effects of interruption timing on human performance.

Generally, a task interruption is defined as a situation in which an ongoing primary task is temporally suspended to perform a secondary task (e.g., Hirsch et al., 2024a). Numerous studies have shown that task interruptions have adverse effects on performance in the primary task (e.g., Altmann et al., 2014; Dodhia and Dismukes, 2009; Radović et al., 2022; see Werner et al., 2015, for a review). To examine these effects, many studies have focused on resumption costs (e.g., Altmann and Trafton, 2004; Blumberg et al., 2015; Monk et al., 2008; Trafton et al., 2003). Resumption costs refer to the difference in the processing time and error rates between the first action after a task interruption relative to the same action in a non-interrupted primary task.

Studies with procedural primary tasks, which comprise a predefined sequence of subtasks, have contributed to the understanding of the cognitive mechanisms underlying the resumption performance (e.g., UNRAVEL task with seven subtasks in Altmann et al., 2017; WORTKLAU task with eight subtasks in Radović and Manzey, 2019). These studies suggest that task interruptions disrupt the memory for past performance rather than attentional resources (e.g., Altmann et al., 2017). Evidence for this notion is provided by the finding that task interruptions have an effect on sequence errors, whereas non-sequence errors are often unaffected by interruptions. Sequence errors occur when one loses track of the subtask sequence and selects a wrong subtask. This leads to the repetition of completed subtasks or the skipping of subtasks that still need to be executed. In contrast, non-sequence errors occur if the correct subtask is selected, but incorrectly executed (for a similar distinction in task switching, see Moretti et al., 2023, 2024).

The disruptive effects of task interruptions are affected by several factors, such as the interruption timing and the interruption duration (see Couffe and Michael, 2017; Trafton and Monk, 2007, for reviews). Studies on the effects of interruption timing on primary-task performance showed that interrupting a primary task between its subtasks is less disruptive for the performance in the primary task than interrupting it during the execution of a subtask (e.g., Bailey and Konstan, 2006; Botvinick and Bylsma, 2005; Cutrell et al., 2000). It has been argued that the mental workload is reduced between subtasks because the cognitive resources allocated to a subtask are momentarily released before they are devoted to the next subtask (Miyata and Norman, 1986). Accordingly, previous studies concluded that low mental workload moments are more suitable for task interruptions than high workload moments. In addition, Bailey and Iqbal (2008) suggested that in contrast to high mental workload moments, at low mental workload moments, fewer cognitive resources are needed to resume the primary task after an interruption.

Importantly, it has been recently shown that the interruption timing can also affect the performance in the secondary task. For instance, Hirsch et al. (2024b) used a procedural primary task consisting of three subtasks. Participants were interrupted before the second or third subtask. During the interruption, they had to perform a single speeded two-choice categorization task. Thus, the interruption duration was not fixed but determined by the processing time of the secondary task. As a result, it was possible to examine the effects of the interruption timing on the performance in the secondary task. The authors observed that reaction times (i.e., RTs) for the secondary task were higher when the interruption was introduced after the first subtask than when it occurred after the second subtask of the primary task. However, this study did not consider differences in mental workload during the primary task. Thus, it is unclear whether, like for primary tasks, mental workload also modulates interruption timing effects in the secondary task.

One limitation of the existing studies examining the effects of mental workload on task-interruption effects is that moments of low mental workload are rather arbitrarily defined. Even though subtask boundaries are likely to be associated with the lowest amount of mental workload, and subtask boundaries seem straightforward to define within a task, task models should consider the cognitive operation during different moments of the task (Adamczyk and Bailey, 2004; Powers and Scerbo, 2023). One instance of such a fine-grained definition is provided by a series of studies using pupil size dilation as a physiological correlate of mental workload (Bailey and Iqbal, 2008; Iqbal and Bailey, 2005; Iqbal and Bailey, 2007). These studies did not only confirm that moments of lower mental workload are best for task interruptions, but they also revealed that the decrease in workload at subtask boundaries is a function of subtask complexity, such that the more complex a subtask, the higher the decrease (Bailey and Iqbal, 2008). These findings indicate that researchers should consider varying degrees of mental workload within subtasks in their task models.

Regarding the effect of task-interruption duration, various studies showed that long task interruptions are more harmful for primary-task performance than short interruptions (e.g., Altmann et al., 2014; Altmann et al., 2017; Foroughi et al., 2016; Hodgetts and Jones, 2006). More specifically, the resumption of the primary task takes longer and is more error-prone after long compared to short task interruptions. This interruption-duration effect is often accounted for by the *memory for goals (MFG) model* (Altmann and Trafton, 2002). According to this model, goals—defined as mental representation of an intention to execute a task—compete for being selected to control behavior, and the goal with the highest activation level is the one that is retrieved. Importantly, task goals decay over time. Since during long interruptions, the primary-task goal can decay more strongly than during short interruptions, it takes more time to reactivate it after a long interruption compared to a short interruption.

To explain task-interruption effects in sequence errors, the notion that place-keeping relies on the interplay of episodic memory and semantic memory has been added to the MFG model (Altmann and Trafton, 2015). Episodic memory comprises a representation of each completed subtask. As these representations decay as a function of time, the episodic representation of the most recently executed subtask has the strongest activation. In contrast, the subtask sequence is represented in semantic memory (see also Hirsch and Koch, 2024, for a similar notion for procedural tasks consisting of two subtasks). This representation includes associative links between subtasks, and activation spreads from the currently relevant subtask to all following subtasks. Importantly, spreading activation decreases with each link. As a result, there is an activation-level ranking of the subtasks to be performed in which the next subtask shows the highest activation level, and the subtask following this subtask a slightly lower activation level, and so on.

To select the next subtask, the most active subtask representation is retrieved from episodic memory which is the representation of the just performed subtask. The retrieved episodic representation is used to specify the next subtask in the predefined subtask sequence. Since within the subtask-sequence representation, spreading activation is strongest for the immediate successor of the retrived subtask, the immediate succesor is retrieved.

The model accounts for sequence errors by assuming that decay is faster for the episodic representations of more recently performed subtasks than for those of older subtasks. Consequently, the relative distance between the activation levels of the episodic representation of the subtask performed before the interruption and the representation of older subtasks is reduced. This can lead to the selection of an incorrect subtask representation.

This brief review of the empirical findings concerning task-interruption effects and their theoretical explanations demonstrates that more research is warranted on two counts. First, the effect of task-interruption timing on primary-task performance has to be examined by using a theory-driven approach to clearly define a task model. Based on such theory-driven approaches, moments of low and high mental workload can be determined more precisely and the effect of interruption timing on performance can be explored more systematically. Second, little is known about the interplay of the interruption timing and the interruption duration.

In the present study, we report an experiment in which we investigated the effects of interruption timing on primary-task performance. The participants performed a procedural primary task consisting of a predefined sequence of six subtasks. The subtasks were speeded two-choice categorization tasks. To specify moments of low and high mental workload, we formulated a theory-driven task model with a hierarchical structure which we derived from the previous literature on sequence chunking and task switching.

From research on sequence chunking, it is known that observers structure events in a hierarchy whose coarse elements, commonly referred to as chunks, are constituted by grouping the fine-grained elements (Zacks et al., 2001b). Also, such a hierarchy was found to correlate with activity within a brain network comprising the frontal eye field, which is involved in event segmentation, so that coarse event boundaries elicited more activity than fine event boundaries (Zacks et al., 2001a).

These findings were used by Adamczyk and Bailey (2004) to build a task model with a hierarchical task structure in which coarse breakpoints (i.e., between chunks) were predicted to elicit smaller resumption costs than fine breakpoints (i.e., within a chunk). This is because after an interruption at coarse breakpoints, participants have to identify the (next) relevant chunk and retrieve it. In contrast, in the case of an interruption at fine breakpoints, participants additionally have to specify their position within the chunk, to resume the primary task. The study by Adamczyk and Bailey (2004) comprised two parts. In the first part, participants were presented with video clips of a primary task and were instructed to determine coarse and fine breakpoints. In the second part, Adamczyk and Bailey (2004) conducted a task-interruption experiment with a new group of participants. In this experiment, interruptions occurred after the coarse and fine-grained breakpoints defined by the participants who had previously watched the task videos. Even though no effects were found on resumption costs, annoyance and frustration were decreased when interruptions occurred in predicted “best” moments.

In the present study, hypotheses regarding the hierarchical structure of our task model were derived from the literature on the backward inhibition paradigm (e.g., Mayr and Keele, 2000; see Koch et al., 2010, for a review). Using this paradigm, we were able to draw on previous studies on backward inhibition, which make clear predictions on task dynamics in sequences of three tasks (e.g., Koch et al., 2006). In the backward-inhibition paradigm, subjects switch between three tasks (A, B, and C) on a trial-by-trial basis. The impact of backward inhibition is detected by comparing performance in the last trial of lag-2 repetition triplets and lag-2 switch triplets. In lag-2 repetition triplets, the same task has to be performed on the first trial and the last trial (e.g., *A*B*A*), whereas in lag-2 switch triplets, the tasks always switch (e.g., *C*B*A*). Performance in the last trial of lag-2 repetition triplets was found to be worse than that in lag-2 switch triplets, reflecting lag-2 repetition costs (e.g., Moretti et al., 2021; see also Hirsch et al., 2017).

From a theoretical view, lag-2 repetition costs are thought to arise due to the lingering inhibition of the task set (i.e., mental representation of a task) to be returned to (see Koch et al., 2010, for a review). Assuming that each task switch requires inhibiting the previously active task set, the reasoning is the following: In an ABA (lag-2 repetition) triplet, the switch from A to B is accomplished by inhibiting task set A. When switching from B to A, task set A is in an inhibited state, and such inhibition must be overcome. In contrast, in a CBA (lag-2 switch) triplet, there is no overcoming of inhibition required, so that the effort associated with the final switch from B to A is not different from the one exerted for a switch from C to B.

Bearing this in mind, it is possible to state that the highest workload is reached when switching from trial lag-1 to trial *n* (i.e., last trial) in a lag-2 repetition triplet, namely when inhibition must be overcome. On the triplet level, we can, thus, assume the following task structure: Switching between the lag-2 and lag-1 trials is associated with the lower mental workload in both lag-2 repetition triplets and lag-2 switch triplets, whereas the switch between lag-1 and *n* trials can be of a comparable workload in a lag-2 switch triplet, or of an increased workload in a lag-2 repetition triplet due to demand to overcome inhibition. Thus, in addition to the cognitive processes required for resuming the third subtask in lag-2 switch triplets, in lag-2 repetition triplets, participants have to overcome the inhibitory aftermath of the relevant subtask when resuming the primary task. As a consequence, resumption costs for the third subtask should be higher in lag-2 repetition sequences than in lag-2 switch sequences.

Note that to posit such a structure between subtask boundaries (i.e., during subtask switches) participants must know in advance which subtask will be requested next. In the present study, a pre-defined sequence of six subtasks consisting of either two lag-2 repetition triplets or two lag-2 switch triplets was given as primary task. Participants were asked to perform the subtask sequence while being occasionally interrupted on different positions, namely before the second, third, or fourth subtask (i.e., during the first or second switch within the first triplet or during the switch from the last subtask of the first triplet to the first subtask of the second triplet).

**Figure 1:**
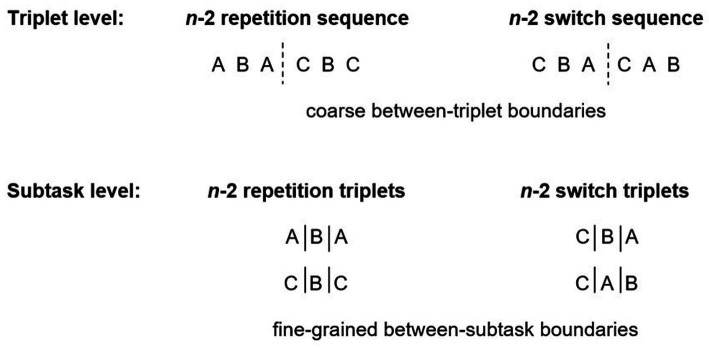
Hierarchical task model with coarse between-triplet boundaries and fine-grained between-subtask boundaries for primary tasks including a *n*-2 repetition sequence (two lag-2 repetition triplets) and for those including a *n*-2 switch sequence (two lag-2 switch triplets).

This approach allows studying task interruptions within the context of a theoretically built task model, in which relationships between subtask boundaries are specified. Also, the use of two triplets renders the task model hierarchical: Fine-grained subtask boundaries can be defined as outlined above, and coarse subtask boundaries should be present between triplets (e.g., ABA–CBC; see Figure 1). This idea is supported by studies employing the exact same structure, suggesting that giving instructions on the task sequence, leads to chunking of the six tasks in two triplets (Koch et al., 2006; see also Radović and Manzey, 2019). As a result, subtasks are not sequentially retrieved from memory, but they are grouped into units, referred to as chunks. Each chunk can be retrieved from memory and executed as a single unit (see Abrahamse et al., 2013, for a similar idea for motor chunking). Note that chunking has been in the focus of numerous studies (Brown and Koch, 2024; Rosenbaum et al., 1987; see Verwey et al., 2015, for a review). However, most of these studies examined chunking at the level of simple key presses (e.g., Verwey and Eikelboom, 2003), whereas the present study addressed chunking at the level of subtasks.

In addition, we aimed to explore how interruption-timing effects are affected by the interruption duration. To this end, we employed interruptions of 2 and 4 s. We decided on relatively short interruptions because Monk et al. (2008) pointed out that differences in the interruption duration across previous studies might have caused inconsistent findings. Studies reporting null effects have often employed overlong interruptions (e.g., 30 and 165 s. in Gillie and Broadbent, 1989). Given that the decay curve proposed in the memory for goal model is a power function, with greater decay occurring in proximity of interruption onset, the model predicts that at long interruption durations the activation level will have reached an asymptotic state.

From these reviewed theory-driven considerations, we derived three hypotheses and examined them in an experiment including a primary task with a predefined sequence of six speeded two-choice categorization tasks and a secondary task requiring speeded vowel-consonant categorizations. First, we predicted resumption costs, reflecting worse performance in a subtask occurring after a task interruption than in the same subtask of a non-interrupted primary task. Second, we hypothesized an interruption-duration effect with higher resumption costs after a long interruption than after a short interruption. Third, we predicted an interruption-timing effect with higher resumption costs for interruptions at moments of high mental workload than for interruptions at moments of low mental workload. More precisely, we expected interruptions before the fourth subtask being the less detrimental due to the chunking of the six subtasks into two triplets, as it would occur at between-subtask boundaries. Interruptions before the second and the third subtasks should result in similar resumption costs in lag-2 switch triplets, but in lag-2 repetition triplets, interruptions before the third subtask should be more disruptive than interruptions before the second subtask. This is because of the need for overcoming inhibition. Moreover, we were interested in the influence of the interruption duration on this interruption-timing effect and in the effect of the interruption timing on the performance in the secondary task. However, it should be noted that the analysis of these two effects was exploratory due to the lack of precise predictions.

## 2 Materials and methods

### 2.1 Participants

Forty-eight psychology students (37 female, 11 male; 42 right-handed and 6 left-handed, *M* = 23.9 years; *SD* = 4.2) with normal or corrected-to-normal vision participated in this experiment and received partial course credit. The sample size was specified based on an a-priori sample size calculation with MorePower 6.0.4 (Campbell and Thompson, 2012). The calculation resulted in a sample size of *N* = 22 per each interruption-duration group, to detect large effects of *η*_p_^2^ = 0.14 with a power of 90%. Due to reasons of counterbalancing, we tested 24 subjects in each group. Note that we chose a large effect size because this effect size is in the range of the effect sizes reported for task-interruption effects (e.g., Altmann et al., 2017; Hirsch et al., 2024a; Monk et al., 2008).

### 2.2 Tasks, stimuli, and responses

The primary task included three different subtasks which unfolded in a pre-defined order of six subtasks. The subtasks were to categorize digits as (A) odd or even, (B) lower or higher than 5, and (C) peripheral (1, 2, 8, and 9) or central (3, 4, 6, and 7) to 5. The stimuli were digit from 1 to 9, excluding 5. Responses were made on a QWERTZ keyboard. Each of the three subtasks was mapped to a different pair of fingers and to different response keys. One subtask was performed with the ring fingers which were mapped to the X- and M-keys, another one with the middle fingers which were mapped to the C- and N-keys, and the last one was performed with the index fingers which were mapped to the V- and B-keys. The secondary task was a letter categorization task in which letters were categorized as vowel (A, E, I, O, and U) or consonant (H, L, M, and N). Responses in the secondary task were provided by pressing the Y or – keys with the little fingers.

All stimuli were presented in black and appeared centrally on a gray background. One stimulus was presented at a time and subtended 7° of visual angle from a viewing distance of 100 cm. At the top of the screen, there was a cue consisting of six letters (e.g., ACBCAB). Each letter represented one of the three subtasks of the pre-defined subtask sequence.

### 2.3 Subtask sequences of the primary task

The subtask sequences for the primary task had either two lag-2 repetition triplets (e.g., AB*A*-CB*C*; i.e., lag-2 repetition sequence) or two lag-2 switch triplets (e.g., CB*A*-CA*B*; i.e., lag-2 switch sequence). In each sequence of six subtasks, all of the three subtasks were represented equally often, and there were no immediate subtask repetitions (see Table 1 for the counterbalancing).

**Table 1:** Sequences employed in the present study.

Lag-2 repetition sequence	Lag-2 switch sequence
ABA CBC	CBA CAB
ACA BCB	BCA BAC
BAB CAC	CAB CBA
BCB ACA	ACB ABC
CAC BAB	BAC BCA
CBC ABA	ABC ACB

Triplets within the sequence are separated by a space for illustration purposes.

Each participant was assigned to a pair of primary tasks, so that they performed a lag-2 repetition sequence and a lag-2 switch sequence. The lag-2 switch sequence was matched to the lag-2 repetition sequence so that both subtask *n* and subtask lag-1 (i.e., 3rd and 2nd subtask) of the first triplet and the subtask lag-2 (i.e., 1st subtask) of the second triplet were identical across the two sequences. For instance, if the lag-2 repetition sequence was A*BA*-*C*BC, the lag-2 switch sequence was C*BA*-*C*AB.

### 2.4 Procedure

The participants were tested individually in a single session. The instructions informed the participants that there will be a primary task consisting of six subtasks which had to be performed in a pre-defined order, and that the primary task will be occasionally interrupted. The instructions emphasized speed and accuracy for both the primary and secondary tasks. All participants performed two different primary tasks, including a lag-2 repetition sequence and a lag-2 switch sequence. The primary task-sequence was varied blockwise with counterbalanced order across participants.

Each trial (i.e., primary task) started with the presentation of a fixation cross. After 2,000 ms, the fixation cross disappeared, and a cue indicating the subtask sequence was displayed. The first digit appeared after a cue-stimulus-interval (CSI) of 600 ms. Between the following five digits, there was a response–stimulus-interval (RSI) of 600 ms, during which the cue remained on the screen. In interrupted trials, the cue disappeared, and a letter was presented as an interruption stimulus. The letter categorization task went on until the time limit of 2 or 4 s was reached. After this interruption task, the cue and the digit for the next subtask, separated by a CSI of 600 ms, were presented.

First, participants performed a block of three non-interrupted trials (i.e., primary tasks) to ensure that they understood the tasks correctly. This block was followed by a practice block for the first primary task. The practice block contained 24 trials, of which 21 were non-interruption trials and three interruption trials. In the case of an error, the German word error (“Fehler”) was displayed for 1,000 ms. Finally, participants performed four experimental blocks, each comprising 24 trials. An experimental block consisted of 18 interruption trials and six non-interruption trials. Only one interruption, if any, could occur on each trial. It occurred before the second, third, or fourth subtask and lasted 2 or 4 s. Thus, task interruptions occurred six times for each of the three interruption positions. This procedure was repeated for the second primary task.

### 2.5 Design

To investigate whether task interruptions impair primary-task performance, we analyzed the independent within-subjects variable *trial typ*e (i.e., interrupted trials vs. non-interrupted trials). More precisely, we contrasted the performance in a subtask after an interruption with the performance in the same subtask in non-interrupted primary tasks. The dependent variables were RTs, non-sequence error rates, and sequence error rates.

To examine the effects of interruption timing and duration on primary-task performance, we used a 3×2×2 mixed design with the within-subject independent variables *interruption position* (i.e., before second, third, vs. fourth subtask) and *subtask sequence* (i.e., lag-2 switch vs. lag-2 repetition sequence). *Interruption duration* (i.e., 2 vs. 4 s) was a between-subjects independent variable. The dependent variables were resumption costs (i.e., difference between a subtask after an interruption and the corresponding subtask in non-interrupted primary tasks) in RTs, non-sequence error rates, and sequence-error rates.

The effects of the interruption timing and duration on the performance in the secondary task were analyzed based on a 2 × 2 mixed design including the independent within-subjects variable *interruption position* (i.e., before second, third, vs. fourth subtask) and the independent between-subjects variable *interruption duration* (i.e., 2 vs. 4 s). The dependent variable was the mean RT for the secondary task in interrupted trials (i.e., trials that included a secondary task). We calculated the mean RT by adding the individual RTs for each categorization task performed during the interruption and dividing this sum by the number of categorization tasks completed during the interruption.

Moreover, we checked whether the lag-2 sequence manipulation resulted in lag-2 repetition costs, an index of an increased mental workload due to the need to overcome lingering inhibition. For this analysis, we used trials with an interruption before the fourth subtask as baseline trials and contrasted performance in the third subtask across lag-2 switch and lag-2 repetition sequences. We measured RTs, sequence errors, and non-sequence errors.

Finally, to explore whether participants divided the six subtasks of the primary task into two chunks consisting of three subtasks, we analyzed the independent within-subjects variable *subtask position*. Basically, we used non-interrupted primary tasks and compared RTs and error rates in the fourth subtask with those in the third subtask and the fifth subtask.

## 3 Results

*T*-tests and analyses of variance (ANOVAs) were run with R (version 4.9.9, R Core Team, 2021; see https://osf.io/9vygs/ for raw data and analysis code). For data analyses, we removed practice blocks, subtasks of interrupted trials which were never preceded by an interruption (i.e., first, fifth, and sixth subtask). Also, we excluded those subtasks belonging to an interruption trial but not occurring right after the interruption (e.g., second and third subtasks in a trial with an interruption before subtask 4).

Moreover, for the RT data analysis, we filtered out subtasks with an error and excluded outliers with RTs over the 95th percentile of the distribution (i.e., condition-wise) (i.e., together 20.1% of the trials). Finally, we assessed the number of “survived” trials for each participant in each condition to ensure that they had at least 10 trials in each cell of the statistical design. Five participants had to be excluded, as they did not satisfy this criterion (i.e., 2 in the group with a short interruption and 3 in the group with a long interruption). Furthermore, data of one participant had to be excluded due to problems with the keyboard mapping, thus resulting in an extremely high error rate (i.e., 74.6%). Thus, our final sample was composed of 42 participants. For the accuracy analyses, we took the arcsine of the square root of the error rate (Laurencelle and Cousineau, 2023) as dependent variable^1^.

### 3.1 Resumption costs in the primary task

**Figure 2:**
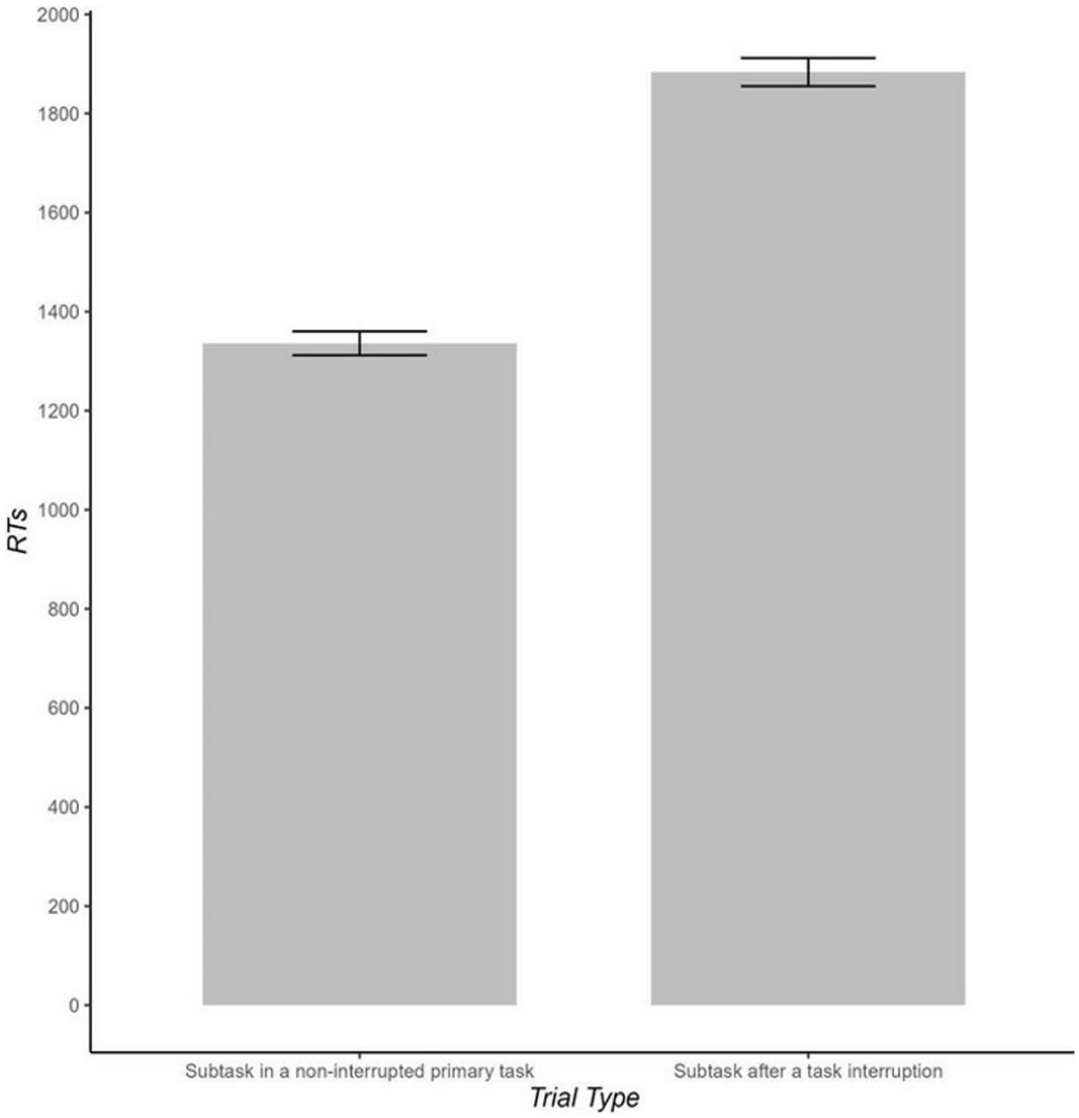
Reaction times (RTs in ms) as a function of trial type (i.e., subtask after a task interruption vs. the same subtask in a non-interrupted primary task). Error bars indicate standard error of the mean.

One-tailed paired *t*-tests showed that RT and sequence-error rates were higher when the subtask followed an interruption than when the same subtask occurred in non-interrupted trials (1,857 ms vs. 1,311 ms and 7.3% vs. 1.5%), *t*(41) = 14.05, *p* < 0.001, *d* = 2.17 for RT and *t*(41) = −9.11, *p* < 0.001, *d* = 1.40 for sequence error rates (see Figure 2 and Table 2). Non-sequence error rates did not differ significantly across interrupted and non-interrupted trials, *t*(41) = 1.14, *p* = 0.262.

**Table 2:** Rates of non-sequence errors and sequence errors (in %; standard errors in parenthesis) as a function of trial type (interrupted vs. non-interrupted trials), subtask sequence (lag-2 repetition sequence vs. lag-2 switch sequence), interruption duration (2 vs. 4 s), and interruption position (before second, third, or fourth subtask).

	Lag-2 repetition sequence			Lag-2 switch sequence		
	Before subtask 2	Before subtask 3	Before subtask 4	Before subtask 2	Before subtask 3	Before subtask 4
Non-interrupted trials						
Non-sequence errors	2.5 (4.3)	2.3 (3.4)	3.3 (5.4)	3.5 (6.0)	2.9 (4.9)	4.1 (7.7)
Sequence errors	3.9 (5.9)	4.7 (7.0)	5.2 (8.0)	3.5 (5.8)	3.7 (6.8)	5.4 (8.2)
Interrupted trials with 2 s						
Non-sequence errors	3.0 (5.5)	2.7 (4.0)	2.8 (4.0)	3.6 (6.0)	3.1 (4.6)	3.5 (5.4)
Resumption cost for non-sequence errors	0.6 (6.2)	−0.6 (2.8)	−1.0 (3.6)	1.3 (4.4)	−0.1 (3.9)	−0.7 (4.4)
Sequence errors	4.2 (6.5)	4.2 (4.7)	4.5 (6.1)	2.6 (6.1)	3.4 (5.9)	5.1 (7.4)
Resumption cost for sequence errors	6.5 (7.5)	2.6 (6.3)	4.5 (8.5)	2.6 (7.7)	4.9 (6.8)	5.8 (10.0)
Interrupted trials with 4 s						
Non-sequence errors	1.9 (2.5)	1.9 (2.7)	3.9 (6.6)	3.5 (6.0)	2.6 (5.3)	4.6 (9.7)
Resumption cost for non-sequence errors	0.3 (3.4)	0.7 (3.2)	−0.3 (3.1)	0.1 (4.9)	−0.6 (5.0)	0.2 (4.2)
Sequence errors	3.5 (5.2)	5.2 (8.9)	6.0 (9.8)	4.4 (5.5)	4.1 (7.7)	5.7 (9.0)
Resumption cost for sequence errors	6.0 (6.1)	8.3 (10.5)	8.7 (12.7)	6.4 (6.9)	6.5 (9.7)	7.0 (12.4)

### 3.2 Effects of interruption timing and duration on primary-task performance

**Figure 3:**
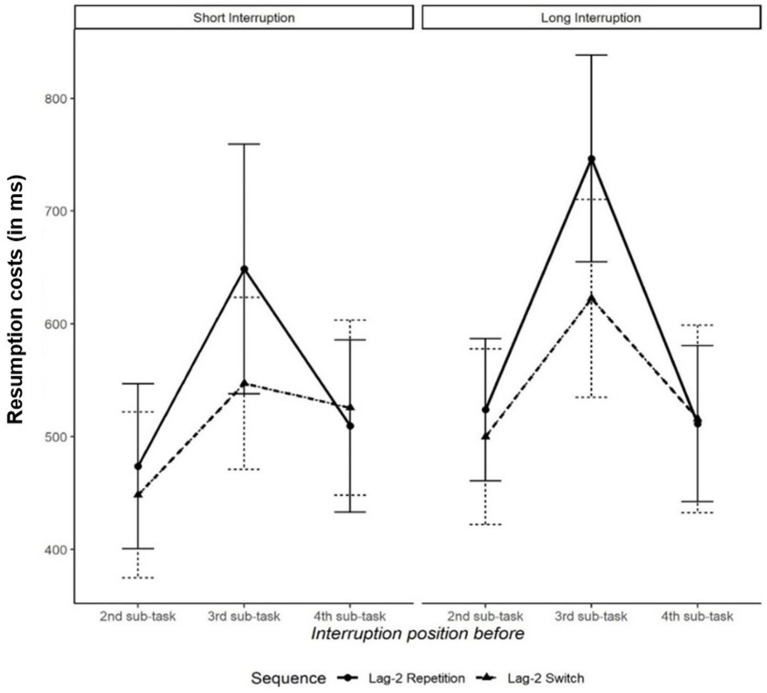
Resumption costs (in ms) as a function of interruption position (before 2nd, 3rd, or 4th subtask), subtask sequence (lag-2 repetition vs. lag-2 switch sequence), and interruption duration (2 or 4 s). Error bars indicate standard error of the mean.

The ANOVA on resumption costs (i.e., difference between the performance in a subtask following an interruption and the performance in the same subtask of a non-interrupted primary task) in the RT data yielded a significant main effect of interruption position, indicating that the most time was needed to resume the primary task after interruptions before the third subtask, followed by interruptions before the fourth subtask and interruption before the second subtask (639, 515, and 485 ms, respectively), *F*(2,80) = 6.12, *p* = 0.003, $\eta_{G}^{2}$ = 0.03. One-tailed paired *t*-tests demonstrated that it took significantly longer to resume the primary task after interruptions occurring before the third subtask than after interruptions presented before the second subtask, *t*(41) = 3.39, *p* = 0.002, *d* = 0.52, and before the fourth subtask, *t*(41) = 2.68, *p* = 0.011, *d* = 0.41. Resumption costs for interruptions before the second and the fourth subtasks did not differ significantly, *t*(41) = −0.64, *p* = 0.524. The main effect of interruption duration and the interaction of interruption position and subtask sequence, both *F*s < 1, were not significant. All other effects were not significant, too, all *F*s < 1.29 and all *p*s > 0.263 (see Figure 3).

The ANOVA on the resumption cost in the non-sequence errors rates revealed no significant effects, all *F*s < 1.41 and all *p*s > 0.251. The ANOVA on the resumption cost in sequence-error rates showed that participants tended to make more sequence errors when resuming a subtask after a long interruption compared to resuming it after a short interruption (4.8% vs. 4.0%; see Table 2). The corresponding main effect of interruption duration was, however, not significant, *F*(1,40) = 3.58, *p* = 0.066, $\eta_{G}^{2}$ = 0.02. The interaction of interruption position and subtask sequence, *F* < 1, and all other effects were not significant, too, all *F*s < 1.78 and all *p*s > 0.177.

### 3.3 Effects of interruption timing and duration on secondary-task performance

**Figure 4:**
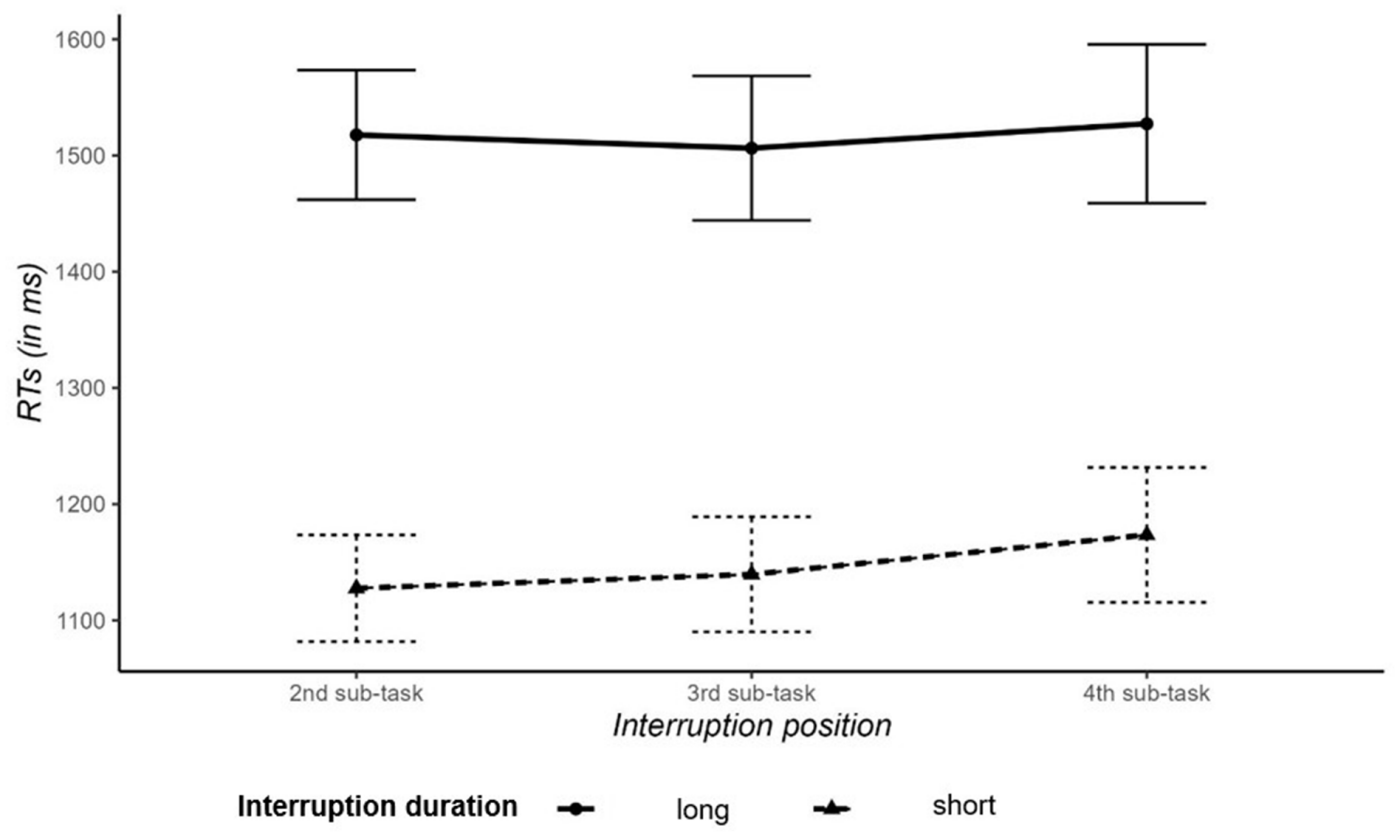
Reaction times (in ms) in secondary-task trials as a function of the interruption position (before 2nd, 3rd, or 4th subtask) and interruption duration (2 or 4 s). Error bars indicate standard error of the mean.

The ANOVA revealed a main effect of interruption duration, indicating that secondary-task trials were performed more slowly for long interruptions than for short interruptions (1,517 ms vs. 1,147 ms), *F*(1,41) = 21.83, *p* < 0.001, $\eta_{G}^{2}$ = 0.31 (see Figure 4). The main effect of interruption position and the interaction of interruption duration and interruption position were not significant, both *F*s < 1.

### 3.4 Lag-2 sequence effect in baseline trials of the primary task

One-tailed paired *t*-tests showed that RT and non-sequence error rates in the third subtask did not significantly differ across lag-2 repetition sequences and lag-2 switch sequences, *t*s < 1 for RT and non-sequence errors. For sequence errors, there were, however, significant lag-2 repetition costs, with more sequence errors in lag-2 repetition sequences than in lag-2 switch sequences (1.6% vs. 0.7%), *t*(43) = 2.31, *p* = 0.025, *d* = 0.35, indicating that participants avoided lag-2 repetitions^2^.

### 3.5 Chunking effects in baseline trials of the primary task

**Figure 5:**
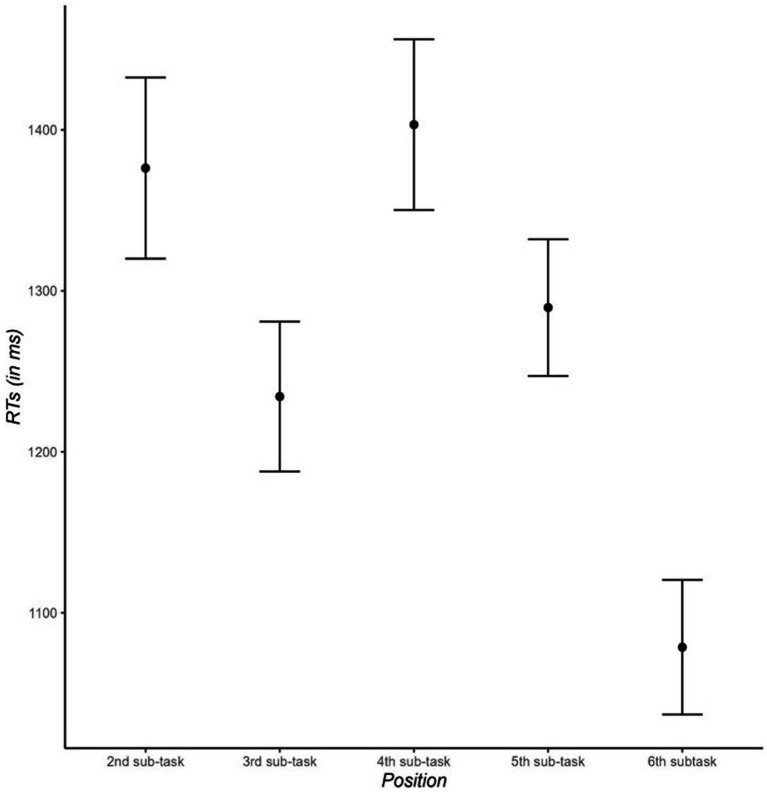
Reaction times (in ms) in the primary task as a function of the subtask (second, third, fourth, fifth, sixth). Error bars indicate standard error of the mean.

A linear model using treatment contrasts and position 4 as the reference category showed that RTs in the fourth subtask were higher than those in third subtasks (1,378 ms vs. 1,199 ms; see Figure 5), *t*(43) = 2.61, *p* = 0.001, *d* = 0.543, and that RTs in the fourth subtask also tended to be higher than those in the fifth subtask (1,379 ms vs. 1,258 ms), *t*(43) = 1.75, *p* = 0.08, *d* = 0.374.

## 4 General discussion

In this study, we defined a task model with a hierarchical structure based on a theory-driven approach to examine how interruption timing modulates the disruptive effects of task interruptions. We found resumption costs, and these costs were affected by the interruption timing. More specifically, resumption costs were higher for task interruptions occurring before the third subtask relative to interruptions before the second and the fourth subtasks. Moreover, in non-interrupted primary tasks, we found worse performance in the fourth subtask than in the third subtask, suggesting that the six subtasks of the primary tasks were chunked in two subtask triplets. Finally, we observed that secondary-task trials were performed more slowly for long interruptions than for short interruptions, but the interruption position did not affect the performance in the secondary task.

### 4.1 Resumption costs

In line with our hypotheses, we observed resumption costs, indicating that task interruptions impair primary-task performance. The cognitive processes involved in the resumption process can be further specified by the findings concerning the resumption accuracy. As indicated by the observation that task interruptions affected the occurrence of sequence errors but had no effect on non-sequence errors, the resumption process seems to rely on memory-based processes rather than on general attentional resources (Altmann et al., 2017). This replicates previous studies (e.g., Altmann and Trafton, 2004; Radović and Manzey, 2022; Ratwani et al., 2006; see, e.g., Hirsch et al., 2022, for a review) and indicates that our primary task is suitable for studying task-interruption effects.

### 4.2 Interruption-duration effect

Several studies found that the harmful effects of task interruptions on primary-task performance are stronger for long than for short task interruptions (e.g., Monk et al., 2008; see, e.g., Hirsch et al., 2022, for a review). This finding suggests that as predicted by the MFG model, the primary task decays as a function of time. As a result, more time is needed to reactivate the primary-task goal after long compared to short task interruptions. The present study showed only a non-significant trend toward higher resumption costs for the long task interruption than for the short task interruption. Note that since we employed a sample size which allowed for the identification of large task-interruption effects, this trend might be related to insufficient power.

Interestingly, there was an interruption-duration effect for the secondary task. That is, secondary-task trials were performed more slowly during long interruptions than during short interruptions. Since, according to the MFG model, the activation of a task decays, once a task is retrieved, there was more time for this decay process during long interruptions than during short interruptions. Further support for this notion comes from task-switching studies examining the within-run slowing effect (e.g., Altmann, 2002; Poljac et al., 2009). These studies showed a gradual increase in RTs across successive task repetitions. The within-run slowing effect indicates that the activation of a task decays as a function of time, even if the task remains relevant for multiple successive trials.

### 4.3 Interruption-timing effect

In the present study, we used primary tasks consisting of two lag-2 repetition triplets or two lag-2 switch triplets and expected greater resumption costs after task interruptions at moments of high mental workload than after task interruptions at moments of low mental workload. Basically, we hypothesized that task interruptions before the fourth subtask, which was located between the first and the second triplet, would be less harmful due to the chunking of the six subtasks of the primary task into two triplets, as it would occur at between-subtask boundaries. Moreover, we predicted that task interruptions before the second and third subtask have comparable effects in lag-2 switch triplets but interruptions before the third subtask are more disruptive for lag-2 repetition triplets, as the workload is higher here due to the need for overcoming inhibition.

We observed an interruption-timing effect. As indicated by resumption costs, task interruptions before the third subtask were more disruptive than those before the second and the fourth subtasks. However, this detrimental effect was not modulated by the lag-2 sequence. In other words, the assumed need to overcome inhibition in lag-2 repetition sequences which should increase the mental workload compared to lag-2 switch sequences did not reinforce the performance decline after task interruptions. Moreover, task interruptions before the fourth subtask did not result in the weakest performance decline. The idea was that the fourth subtask is the first subtask of a new chunk and that task interruptions before the fourth subtask represent an interruption between chunks. Evidence for the notion that the subtasks were chunked into two triplets was provided by the finding that the performance in baseline trials was worse in the fourth subtask than in the third subtask. The decline in the fourth subtask might reflect planning processes related to the next chunk.

Note that since we observed lag-2 repetition costs in the sequence errors of baseline trials, we assume that the mental workload was increased in the last subtask of lag-2 repetition triplets. The absence of a modulation of the interruption-timing effect by the specific subtask sequence might be attributable to different factors. For instance, inhibition might decay as a function of time, and there was more time for the decay in trials with a task interruption than without it. Thus, no lingering inhibition might be left after secondary-task completion. This might have decreased the mental workload associated with the need to overcome inhibition in lag-2 repetition sequences. Note, however, that task-switching studies provide evidence that task inhibition appears to be quite persistent and not to decay quickly as a function of time (e.g., Gade and Koch, 2005). Moreover, to the best of our knowledge, there is so far no other study which examined lag-2 repetition costs in sequence errors. The observed lag-2 repetition cost in the sequence errors of the present study indicates that participants avoided to return to a subtask that was already performed as first subtask. A possible reason for this might be that due to backward inhibition, this subtask was less activated than the other subtasks and, therefore, there were more subtask selection errors. However, more research is warranted to test this assumption.

Even though the present study does not allow us to determine whether there was enough lingering inhibition of the third subtask after a task interruption, it suggests that participants chunked the six subtasks into two subtask triplets, and that interruptions at the end of a subtask chunk are more harmful for the primary-task performance than those between chunks or at the beginning of a chunk (i.e., before the second subtask of the first chunk). This finding cannot be accounted for by the MFG and its extension to procedural primary tasks. According to this model, the episodic representation of the recently performed subtask is employed to specify the next subtask in the pre-defined subtask sequence. The more subtasks participants completed, the more difficult it should be to resume the primary task. This is because with each completed subtask, there is an additional episodic subtask representation, increasing the interference between these representations and, thus, hampering the identification of the last subtask performed before the interruption. From these notions, the prediction can be derived that resumption costs should increase with each completed subtask. Applied to the present study, resumption costs should be lowest for the second subtask, followed by the third subtask, and finally by the fourth subtask.

However, in contrast to this prediction, we observed that resumption costs were higher for the third subtask than for the fourth subtask. A potential explanation for this data pattern might be that in addition to the representations of just completed subtasks, the episodic memory stores representations of just completed subtask chunks. Note that in baseline trials, we observed evidence that participants divided the subtask sequence into two chunks consisting of three tasks (i.e., worse baseline performance in the fourth subtask than in the third subtask; see Radović and Manzey, 2019, for similar findings).

Based on the notion of episodic chunk representations, greater resumption costs for the third subtask than for the fourth subtask can be explained as follows: When resuming the third subtask, there should be at least two subtask representations in episodic memory, and when resuming the fourth subtask, there should be three episodic subtask representations. Consequently, it is conceivable that, as predicted by the MFG model, there was stronger interference at the level of episodic subtask representations when resuming the fourth subtask compared to the third subtask. However, contrary to the third subtask, the fourth subtask might have been primed by the episodic representation of the chunk just completed before the task interruption. This beneficial priming effect at the level of episodic chunk representations might have reduced the interference at the level of episodic subtask representations. This is because at the level of chunks, there should be only one episodic chunk representation, and therefore, between-chunk interference should be low.

Greater resumption costs for the third subtask than for the second subtask can be accounted for by interference at the level of episodic subtask representations, without referring to episodic chunk representations. This is because both subtasks are part of the first chunk. When resuming the second subtask, there should be only one episodic subtask representation and thus less interference between episodic subtask representations than when there are two episodic representations during the resumption of the third subtask.

A further explanation for the observed findings can be derived from the reconstruction-model often used in applied task-interruption research (Salvucci, 2010; Salvucci et al., 2009). The model posits that a reconstruction of the task context occurs, if subjects fail to retrieve the goal of the just completed subtask. More specifically, it is assumed that subjects re-encode the task environment to reconstruct the task context immediately before the interruption. Thus, they re-encode information necessary to identify the next step to be performed (Salvucci, 2010). For instance, if warehouse workers are interrupted by a mobile robot during order picking, workers may scan their checklist of orders and continue picking the next product that has not been checked-off on the list. In the case of interruptions right at the start of their order, warehouse workers have to check fewer boxes on their checklist than in cases of interruption near the end of the order, resulting in a faster reconstruction of the task context and resumption of the primary task.

Applying this model to our finding of longer resumption times for the third subtask as compared to the second subtask, it can be argued that in the case of interruptions before the third subtask, subjects have to reconstruct more subtasks already performed than in the case of interruptions before the second subtask. This difference might result in longer resumption times for interruptions before the third than before the second subtask.

The reconstruction model might also account for why resuming the fourth subtask is less costly than resuming the third subtask. In the case of an interruption before the third subtask, subjects have to re-encode the information that they have performed two subtasks before the interruption, whereas in the case of an interruption before the second triplet, they have to re-encode the information that they have already performed the first triplet, probably without specifying the individual subtasks of this triplet. Thus, across these interruption positions, there is a different amount of information that has to be reconstructed.

## 5 Summary and conclusion

This study suggests that task interruptions at the end of a subtask chunk were more harmful than those at the beginning of a chunk or between two chunks. A possible explanation for this finding is that in addition to the representation of just completed subtasks, the episodic memory stores representations of completed chunks. Both these representations can facilitate the resumption process by priming the following subtask. This finding can be used to expand current models on task interruption.

## Data Availability

The datasets presented in this study can be found in online repositories. The names of the repository/repositories and accession number(s) can be found: https://osf.io/9vygs/.

## Footnotes

^1^Note that analyzing the data without excluding participants revealed the same data pattern.

^2^The reason for the difference in dfs is that the data cleaning is done slightly differently for the analysis of lag-2 repetition costs than for the previous analyses. For example, in the lag-2 repetition costs analysis, the performance in the third subtask is only analyzed when the entire triplet was performed correctly. Applying a different data trimming procedure resulted in the exclusion of less participants.
